# A Review: Development of a Synthetic Lactoferrin Biological System

**DOI:** 10.34133/bdr.0040

**Published:** 2024-08-20

**Authors:** Kun Liu, Zhen Tong, Xuanqi Zhang, Meryem Dahmani, Ming Zhao, Mengkai Hu, Xiangfei Li, Zhenglian Xue

**Affiliations:** Anhui Engineering Laboratory for Industrial Microbiology Molecular Breeding, College of Biology and Food Engineering, Anhui Polytechnic University, Wuhu 241000, China.

## Abstract

Lactoferrin is an iron-binding glycoprotein with antibacterial, antitumor, and immunomodulatory functions derived from milk and mucosal secretions. Lactoferrin is used in various products, such as infant formula milk powder, nutritional supplements, and cosmetics. Researchers have developed new technologies to produce lactoferrin because there are limitations in the separation and purification of lactoferrin from milk that cannot compensate for the market demand. Therefore, synthetic systems of lactoferrin have been developed with the development of genetic engineering, and the structure of lactoferrin expressed in heterologous systems is very similar to that of natural lactoferrin. The structure and functions of lactoferrin and the design and construction of synthetic lactoferrin biological systems, especially microbial synthetic systems, including prokaryotic and eukaryotic host-expression systems, are described. On the basis of these results, we summarize the challenges and solutions for constructing systems of high-yield lactoferrin. The development directions of recombinant lactoferrin are discussed in this review. Overall, the design and development of these synthetic biological systems have allowed us to explore the great potential of the industrial large-scale preparation of lactoferrin.

## Introduction

Lactoferrin (LF) is a multifunctional glycoprotein of the transferrin family and can be found in a variety of body fluids, such as milk, saliva, urine, and tears [1]. LF is naturally expressed in cow milk at an average concentration of 0.1 to 0.2 g/l, but bovine colostrum contains approximately 1.5 g/l, and 2 to 4 g/l is expressed in human milk, whereas 6 to 8 g/l is in colostrum [2,3]. Because lacto refers to “derived from milk” and ferrin refers to “iron-binding protein”, LF was named for its ability to bind to iron, resulting in a distinctive red color. Scientists have been interested in LF since its discovery in the 1930s because of its diverse biological properties, which extended beyond its primary function of binding iron [4]. LF plays roles in the immune response, antimicrobial activity, anti-inflammatory effects, and other biological roles because of its biological characteristics. LF possesses distinct advantages in terms of antibacterial properties by sequestering iron, which is an essential nutrient for bacterial and fungal growth [5,6]. LF acts as a first line of defense against pathogens, effectively disrupting the integrity of cell membranes and leading to the inhibition of the proliferation of microorganisms by limiting the availability of iron, which contributes to the innate immune response. LF also exerts antiviral activity against a variety of viruses [7,8]. In addition, LF interacts with immune cells, such as macrophages and lymphocytes, by influencing their function and boosting the immune response, and has anti-inflammatory properties that aid in the resolution of inflammation in various tissues [9]. LF regulates inflammatory mediator activity and participates in tissue repair processes, highlighting its potential in the treatment of inflammatory disorders, including inflammatory bowel disease, arthritis, and respiratory ailments, and it is also a bioactive component of human milk that contributes to infant health and development. Therefore, LF has a wide range of applications in the food, cosmetic, and pharmaceutical industries.

However, the LF obtained from the separation and purification of milk cannot compensate for the market demand [10–12]. Therefore, technologies for the preparation of LF are urgently needed. For example, microorganisms can synthesize LF via recombinant gene technology. The development of synthetic biological systems [13–18] has increased the feasibility of using microorganisms to prepare large amounts of LF. LF from different species has been successfully expressed in common prokaryotic and eukaryotic systems and other expression systems. In this review, we describe the biological activity of LF, the design and construction of LF expression systems, and some of the LF applications in the biological field, including the associated challenges, solutions, and opportunities.

## Structure and Functions

### Structure of LF

**Fig. 1.**
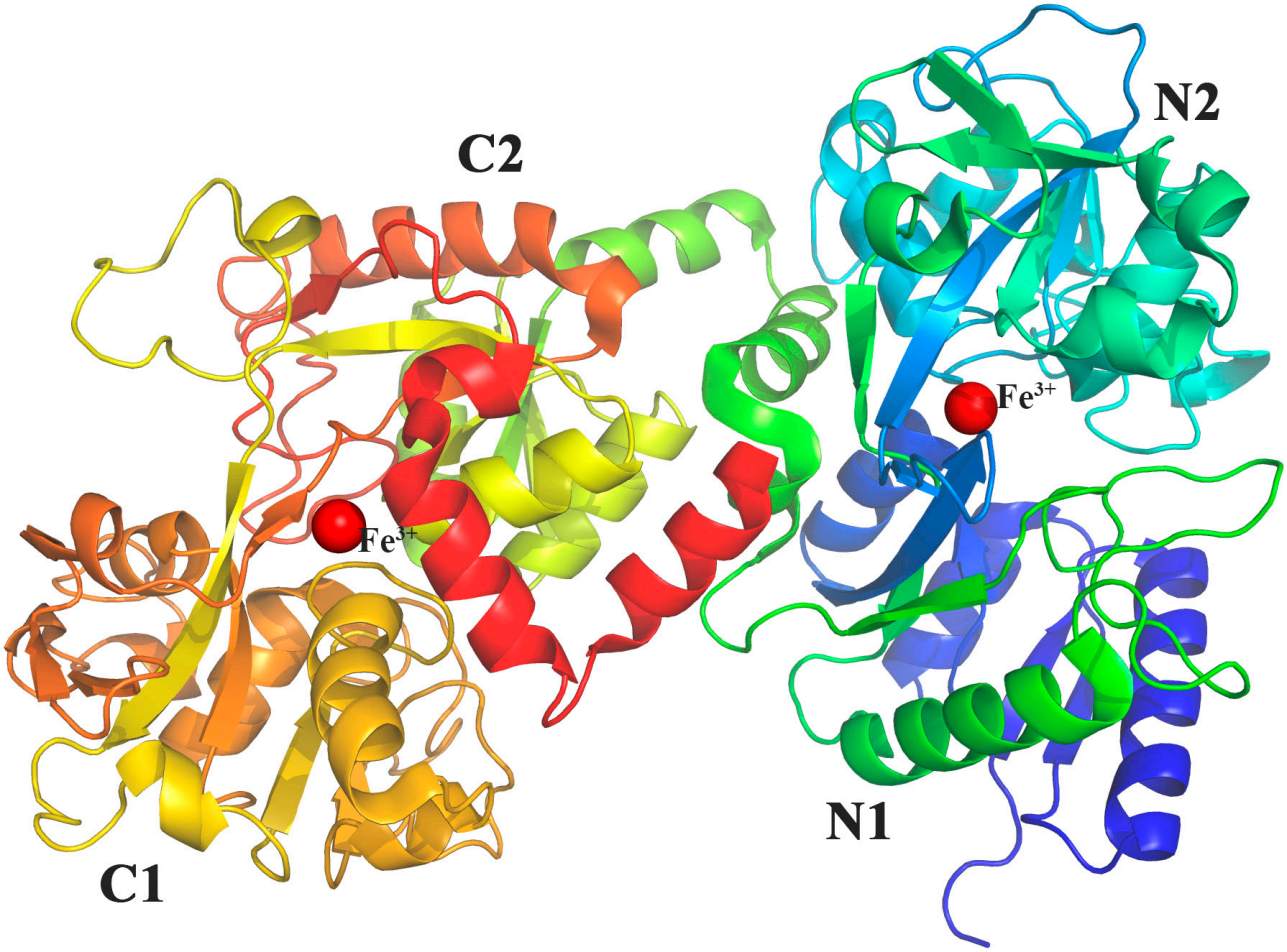
Structure of human LF (PDB: 1FCK). The tertiary structure of LF is a polypeptide chain that folds based on the secondary structure to form 2 very similar and symmetrical spherical sheets, namely, the N lobe and the C lobe. The lobes can be divided into 2 subdomains (N1 and N2, C1 and C2) that have similar sizes.

LF is approximately 700 amino acids long and has a molecular weight of approximately 80 kDa [19,20]. The polypeptide chain is attached to 2 polysaccharide side chains, which are primarily composed of galactose, mannose, N-acetylgalactosamine, fucose, and sialic acid, among other components [21–23]. The folding of the polypeptide chain results in 2 spheres in its 3-dimensional conformation (Fig. 1). The leaf-like, N-leaf, and C-leaf structures have approximately 40% sequence agreement and are linked by α-helices. In the case of human LF 1FCK [24], the N and C lobes are divided into 2 domains, and the fissure between the domains serves as the binding site for Fe^3+^, which is required for binding to anions such as CO_3_^2−^ in combination [25]. The 2 lobes of LF can operate separately, and the iron-binding site on the N-terminal lobe is more acid-labile and thermodynamically less stable than that on the C-terminal lobe [6,26–28]. Therefore, the thermostability of LF can be increased by adding more disulfide bridges to the N-terminal lobe or by replacing it [29–32]. The amino acid sequence of bovine LF (bLF) is ~69% identical to that of human LF, and their secondary structure and 3-dimensional conformation are nearly identical, with only minor differences in the relative positions of the N and C lobes and the degree of closure of the 2 domains within each. The sequence of LF derived from mammals, cattle, sheep, goats, pigs, horses, mice, buffaloes, and camels has also been elucidated [2]. For example, human and cattle LF contain 691 and 696 amino acids, respectively [29]. However, we found that mature LF in human milk does not have an N-terminal signal peptide, which deserves attention. The PDB (Protein Data Bank) database also includes LF structures from rats, horses, pigs, goats, sheep, buffalo, yaks, and camels. LF content varies importantly between different mammalian species, and there have been few reports of the comparative biochemical properties of LF from different sources.

LF is a glycoprotein because of the N-linked glycosylation of asparagine residues in the Asn-X-Thr/Ser tripeptide. On the basis of the amino acid sequence, it was predicted that human LF has 4 potential glycosylation sites, but only 2 of them are attached to glycan chains. bLFLF has 5 potential glycosylation sites, but only 3 are attached to glycan chains [21]. Glycan chains have no effect on the spatial conformation of LF or its biological activity. Although glycosylation has no direct effect on LF, its resistance to trypsin degradation increases, resulting in improved structural stability [33]. Natural LF from mammals has complex glycan chains, whereas recombinantly expressed LF can be glycosylated or not, depending on the expression system and culture conditions. The glycosylation intensity of *Saccharomyces cerevisiae* is greater than that of *Pichia pastoris*, but *Escherichia coli* has a very low level of glycosylation. A suitable expression host system is the key for obtaining high-stability LF.

### Functions of LF

LF has several defense mechanisms against pathogens, including the capacity to fend off bacteria, fungi, viruses, and parasites [5–8]. In addition, LF promotes osteoblast proliferation, immune regulation, anticancer activity, and antioxidant properties [9].

### The effects of LF on viral agents

**Fig. 2.**
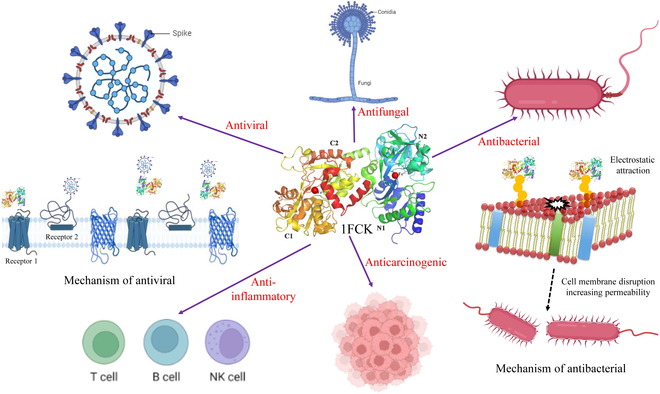
The functions and mechanism of action of LF. Strong antibacterial activity against bacteria, fungi, yeasts, and viruses is given to LF by its broad-spectrum antibacterial effect. The LF compound also enhances the cell–cell interaction and stimulates the proliferation of polymorphonuclear leukocytes and natural killer (NK) cells, thereby enhancing the immune system.

LF is resistant to RNA and DNA viruses that infect humans and other animals [34,35]. Many mechanisms have been proposed to explain the antiviral effects of LF, but the mechanisms described below are the most widely accepted by researchers. LF acts as a barrier to early infection by poliovirus, herpes simplex virus, cytomegalovirus, and human immunodeficiency virus [36–39]. LF interacts directly with virus particles to prevent them from becoming toxic or from replicating in host cells, such as in the case of rotamorphosis and hepatitis C virus [40]. LF can attach itself to rotavirus particles, preventing them from adhering to target cells and causing infection (Fig. 2). In addition, LF can also prevent the synthesis of antigens during rotavirus infection, meaning that it can continue to have an antiviral effect even after the virus has penetrated target cells. LF binds to and blocks glycosaminoglycan virus receptors, especially heparan sulfate, thereby preventing the virus from entering the host cell and preventing infection (Fig. 2). Overall, iron-deficient LF is generally more effective against viruses, but iron-saturated forms can also be involved, indicating that iron saturation may not be the deciding factor [41].

### Effects of LF on bacteria

LF inhibits bacterial growth by binding to Fe^3+^. LF competitively chelates Fe^3+^ with high affinity to inhibit the growth of iron-requiring bacteria, such as *E. coli*, *Streptococcus mutans*, and *Vibrio cholerae,* as well as the formation of biofilms of the pathogenic bacteria *Pseudomonas aeruginosa* [42]. Higher LF antibacterial activity is associated with a lower iron-requiring level of bacteria. Iron scrambling, however, has no complete antibacterial effect and only slows bacterial growth and reproduction [43]. Therefore, iron supplementation eliminates this effect. LF has direct bactericidal activity due to its strong antibacterial influence. In fact, the addition of Fe^3+^ to the bacterial medium significantly increases the production of LF (unpublished). LF connects to subsurface proteins on the outer membrane of gram-negative bacteria that have lipopolysaccharide (LPS) in their cell wall to release LPS (Fig. 2). This increases the permeability of the outer membrane to hydrophobic molecules, which make it easier for lysozyme and other antimicrobial molecules to enter the cell and kill the bacteria [44]. In addition, positively charged N-leaf structures combine with negatively charged teichoic acid in gram-positive bacteria by electrostatic interactions [45]. This process disrupts the nonpolar environment, alters the permeability of the membrane, and ultimately results in the death of the bacteria from leakage of internal components.

### Effects of LF on fungi

LF inhibits or eliminates a range of fungi, such as molds and yeasts. Fernandes et al. [46] assessed the antifungal activity of bLF against 24 molds and 22 yeasts as well as how well it works in combination with 6 common antifungal medications. The experiments revealed that bLF consistently and broadly inhibited all yeasts tested but inhibited only 4 molds. The inhibition of yeast activity was affected by iron saturation. Therefore, we speculate that the addition of metal ions can reduce the toxicity of LF to the expressing host yeast. This speculation may provide a new idea for obtaining high-yield LF. In addition, amphotericin B and bLF demonstrate high synergy against yeasts because they induce pore formation, hyphal thinning, and cell collapse.

In addition to the above fungi, LF also causes the common conditionally pathogenic microorganism *Candida albicans* to undergo regulatory cell death. *C. albicans* cells can swell, leak their contents, or have a depressed or cracked surface before they finally die according to scanning electron microscopy. The release of K^+^ through ion channels leads to changes in cell osmotic pressure [47]. This causes the cells to shrink, promoting apoptosis, but the permeability of the cell membrane remains unchanged over a short period of time. Furthermore, cytoplasmic acidification acts as the first transduction signal to control the cell death pathway, which is caused by LF-mediated inhibition of Pma1p H^+^-adenosine triphosphatase activity [48]. In addition, phosphatidylserine externalization, nuclear chromatin coagulation, DNA degradation, and increased reactive oxygen species (ROS) are features of the LF-induced apoptosis-like phenotype of *C. albicans* [47]. The mechanism of apoptosis was investigated in the *S. cerevisiae* model, and the results suggested that LF can lead to mitochondrial dysfunction associated with the accumulation of ROS and the release of cytochrome c [49]. These results also suggested that mitochondrial energy metabolism is important for the killing efficiency of LF.

## Synthetic Biological System of LF

The LF obtained from the separation and purification of milk cannot compensate for the market demand. The difficulty in obtaining LF can be resolved by using synthetic biological expression systems to produce LF on an industrial scale. This solution can also overcome yield constraints resulting from the scale and industrial structure of animal husbandry. Through host–organism interactions and genetic engineering, these systems enable the controlled synthesis of LF, opening the door to potential uses in food, pharmaceutical, and other industries. A thorough grasp of the transformative potential of LF in industrial domains is achieved by examining the nuances of expression host selection, gene expression design, molecular weight, yield, and LF characteristics (Table 1).

**Table.** Expression of LF and its peptide in various organisms

Organism	LF origin	Expression system	Molecular weight (kDa)	Yield (mg/l)	Characteristics	Reference
Bacteria	*Escherichia coli*	Human LF	pET28a(+)	39	700	Immunoreactive, ability to inhibit breast cancer cell	[53]
		Bovine LF	pET28a(+)	96	15.3	Antibacterial activity	[54]
		Mouse LF	pET28a(+)	40.9	20	Antibacterial activity	[52]
		Mouse LF	pET28a(+)	87.3	17	Antimicrobial activity	[52]
		Bovine LF	Fusion of Lfc and anionic protein genes	80	60	Antimicrobial activity	[75]
	*Rhodococcus erythropolis*	Bovine LF C lobe	pTipLCH1.2	38	3.6	Antiviral activity	[76]
	*Lactobacillus casei*	Human LF	pSD	78	10.6	Antimicrobial activity	[56]
	*Bacillus subtilis*	Human LF	pMA0911	Not reported	16.5	Antimicrobial activity	[25]
Yeasts	*Saccharomyces cerevisiae*	Human LF	pCGY1444 Chelatin promoter	82	2.0	Antimicrobial activity, ability to bind Fe^3+^	[65]
		Equine LF	pPIC9K	80	40	Folded properly, ability to bind Fe^3+^	[66]
	*Pichia pastoris*	Human LF	pPIC3.5K	80	115	Affinity, toxicity, and antiviral activity	[77]
	Bovine LF	pPICZɑA, hybrid signal peptide	10	193.9	Antimicrobial activity	[6]
	Bovine LF	pPIC9K harboring *blf* with promoter P_GAP_	76	824.93	Antimicrobial activity	[78]
	Human LF	CRISPR-Cas9 AOX1 promoter	80	759-870	Antimicrobial activity, ability to bind Fe^3+^	This study (unpublished)
	*Pichia methanolica*	Bovine LF C lobe	pMETα A, α-secretion factor, methanol-inducible AUG1 promoter	Not reported	120	Antimicrobial activity	[79]
	*Komagatella pastoris*	Tibetan sheep LF	pPICZαA	76	60	Antimicrobial activity, ability to bind Fe^3+^	[68]
	Bovine LF	pJ902	80	3500	Antimicrobial activity	[67]
Mold	*Aspergillus nidulans*	Human LF	pAL3hLFT	Not reported	5	Ability to bind Fe^3+^	[70]
	*Aspergillus oryzae*	Human LF	PAhLFG α-amylase promoter of *A. oryzae*	78	25	Immunoreactive, ability to bind Fe^3+^	[20]
	*Aspergillus awamori*	Human LF	pPLF-19 Fusion with the glucoamylase gene	78	2000	Antimicrobial activity, ability to bind Fe^3+^	[71]
Cell lines	CHO	Human LF	pTT5	80	>200	Antimicrobial activity, analysis of structure	[72]
	BHK	Human LF	pNUT	Not reported	20	Ability to bind Fe^3+^	[80]

### Bacteria as hosts to express LF

*E. coli* is one of the most popular bacteria for heterologous LF expression. The well-established genetics of *E. coli* as well as its quick growth and simple manipulation are the reasons behind its popularity, especially the BL21 [14], ArcticExpress, and Rosetta strains [50]. BL21 is selected because it is a reliable and effective host system for heterologous protein expression [51]. BL21 (DE3) was utilized to express and obtain LF and its N-leaf moieties from Kunming mice [52]. Another study by Hu et al. [53] employed BL21 (DE3) for the expression of the C-leaf fraction of human LF and obtained a 700 mg/l C-leaf fraction. However, the inclusion bodies suggested that the expressed C-leaf fraction aggregated into insoluble structures within the bacterial cells. This result is also a common phenomenon when *E. coli* expresses heterologous proteins, especially proteins from higher animals. Fortunately, chaperones are used in *E. coli* as functional proteins to aid in the correct folding of heterologous proteins and reduce the amount of inclusion bodies. To increase expression levels and solubility, a fusion protein made of bLF and thioredoxin was expressed in *E. coli* [54]. In addition, thioredoxin was fused to bLF, which was successfully expressed in BL21 (DE3) cells, and this purified fusion bLF was active. Notably, the fused thioredoxin caused bLF to have a higher molecular weight (96 kDa) than the native protein. The fused thioredoxin had a pI (isoelectric point) of 5.2, which was lower than the native pI of 8.65 [54]. This change in charge may lessen the net charge and conceal the toxicity of mature basic LF, but it may be used as a new antigen rather than as natural LF in the medical field. In addition, cysteine protease domains from *V. cholerae* have been inserted as autocleaving enzyme tags, or different heterologous fusion proteins have been developed in *E. coli* to reduce their toxicity to the host [25]. However, it is crucial to note that *E. coli* lacks the machinery necessary for some posttranslational modifications, and if glycosylation is important, these differences may have an impact on the functions of synthetically expressed LF. The cell-free protein synthesis system, as a technical core of synthetic biology, can simulate the transcription and translation process in an in vitro open environment without a complete living cell. This may open a new path for the large-scale synthesis of LF in vitro [16,17]. Overall, 3 main obstacles prevent *E. coli* from expressing LF or its antimicrobial peptides, as protease activity has the potential to harm recombinant proteins, freshly synthesized proteins are harmful to host bacteria, and hosts lack effective posttranslational modifications.

Other bacteria that have been explored for heterologous expression of LF include *Bacillus species*, specifically *B. subtilis*, which have been employed for LF expression. The pMA0911 plasmid with the P_veg_ promoter offers advantages such as efficient secretion of 16.5 mg/l LF into the culture medium [25]. *Pseudomonas fluorescens* is another bacterium that has been investigated for its potential to express and secrete functional LF [55]. However, there are currently no reports of successful secretion and expression of LF in *P. fluorescens*. *Lactococcus lactis*, a GRAS lactic acid bacterium, can adapt to iron-deficient environments, and the presence of LF does not inhibit its growth ability by chelating iron ions. However, *L. lactis* was used for the expression of recombinant LF to obtain only 10.6 mg/l LF [56]. The genetic background of *L. lactis* is not very clear, which may be the reason for the low level of expression. Every bacterial host has advantages and disadvantages. The decision to select a host is based on various factors, including the intended use of the expressed LF, the required protein folding, the desired posttranslational modifications, and the time of the growth cycle. These properties should be considered to guarantee successful heterologous expression and functional activity of LF.

### Yeast as a host to express LF

Yeast, as a type of host that expresses LF, involves a distinct set of considerations compared with bacterial expression systems. Yeast has the function of posttranslational modification, and the transcription machinery of yeast is used to produce LF by introducing the gene encoding LF. Therefore, yeast is widely used for the expression of heterologously modified proteins. Compared with *P. pastoris, S. cerevisiae* has a stronger ability to glycosylate heterologous proteins. Therefore, *P. pastoris* is more suitable for expressing proteins with fewer glycosylation sites, such as LF. The promoter of alcohol oxidase 1 (AOX1) plays a crucial role in controlling the expression levels of proteins in *P. pastoris*. Alternative promoters, such as the glyceraldehyde-3-phosphate dehydrogenase (GAP) and phosphoglycerate kinase (PGK) promoters, may also be used to fine-tune expression levels on the basis of the specific requirements of the expression system [57,58]. Different process conditions affect the regulation of the AOX1 promoter. Three conditions during fermentation were found to be highly important for the activity of the AOX1 promoter: oxygen-limited, methanol-limited, and switched feeding of carbon sources (such as glucose and methanol) under carbon-limited conditions [59]. In other words, AOX1 activity can be activated only when the medium does not contain sufficient glucose, methanol, or oxygen. Therefore, the regulation of fermentation conditions is very important for increasing the expression level of heterologous proteins. In addition, many genetic and physiological variables, including codon optimization, the number and type of gene copies, translation signals, signal peptides, the coexpression of chaperone proteins, and the ultimate fate of proteins, can affect the levels of heterologous proteins that accumulate in yeast. A signal sequence on a foreign protein was used to direct it to the secretory pathway for it to be secreted into the medium [6,60,61]. This foreign protein was released into the culture media without requiring cell lysis. Protein secretion was facilitated, and the yield was increased by signal peptides, such as the α-factor signal sequence from *S. cerevisiae*. Furthermore, rational design or directed evolution can be used to identify alternative signal peptides with increased secretion efficiency [62]. Chaperone proteins, such as protein disulfide isomerase and heat shock proteins, help correctly fold proteins and prevent them from aggregating. Coexpressing chaperone proteins with LF increases the production of correctly folded and biologically active LF in *P. pastoris* [63,64].

**Fig. 3.**
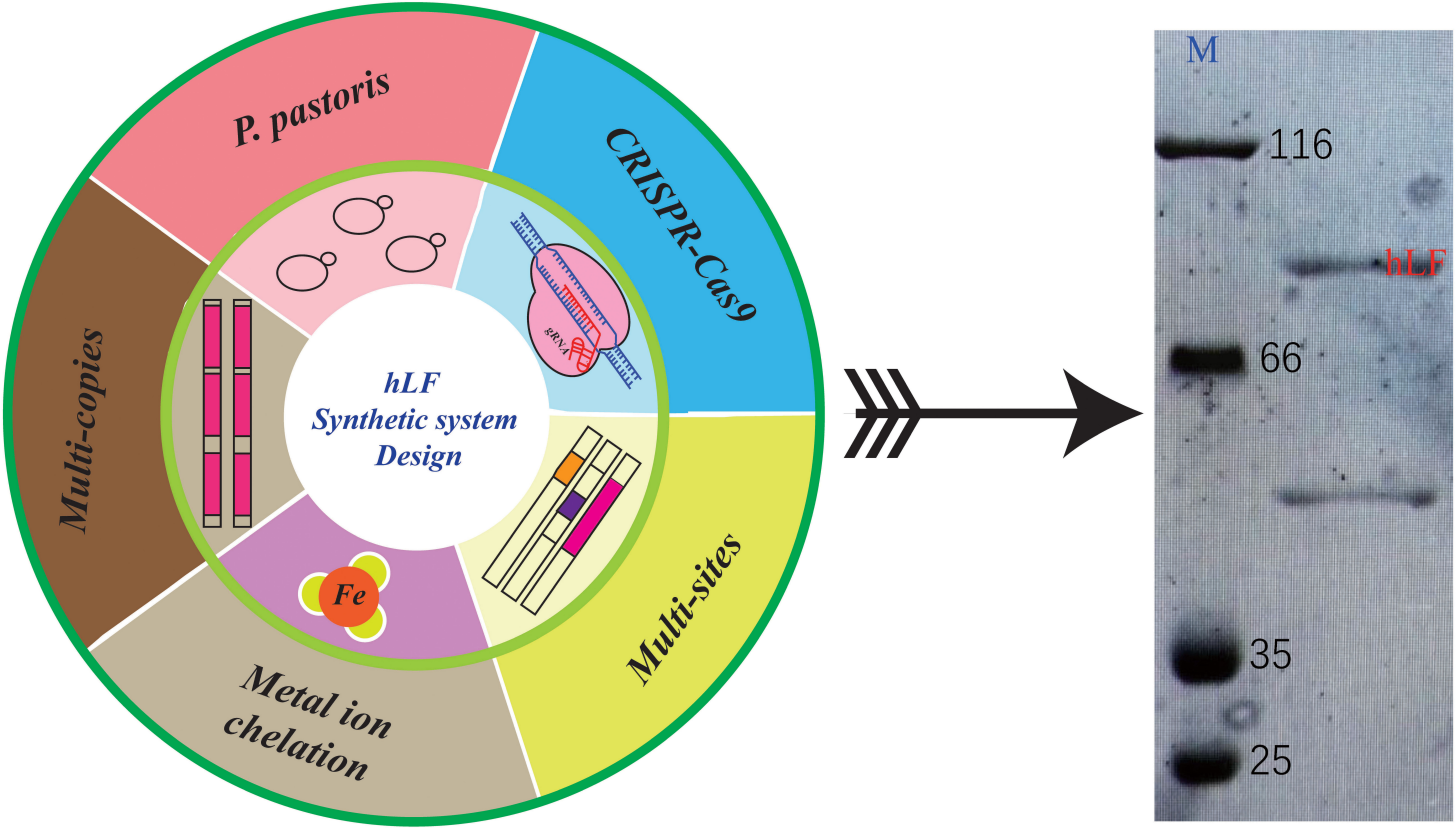
The strategies of multi-copy integration and metal ion chelation were used to obtain high-level expression of hLF.

The LF-fused invertase signal peptide was secreted extracellularly at 1.5 to 2 mg/l in the yeast expression system, while some LF remained within the cell [65]. The results demonstrate the glycosylated and Fe^2+^- and Cu^2+^-binding capabilities of the secreted LF. However, the expression level of the product was much lower in the large-scale culture than it was in the previous test, leading the authors to hypothesize that the toxicity of LF to yeast cells might make stabilizing the expression level difficult. To solve this problem, a high-yield human LF yeast was constructed through CRISPR-Cas9; 6 copies of *hlf* were integrated into different sites of the genome, and the toxicity of LF was reduced by supplementation with metal ions. Finally, the secretion of LF in the fermented supernatant reached 759 to 870 mg/l (Table 1 and Fig. 3). In addition to *P. pastoris*, reports have successfully demonstrated that *S. cerevisiae* can produce foreign glycoproteins, such as the surface antigens of hepatitis B virus, interferons, and monoclonal antibodies. Since LF is a glycoprotein, *S. cerevisiae* was first selected as an appropriate expression system for investigating the connection between the structure and function of human LF [65]. Two plasmids, pRLl and pLFISlO, were created to produce human LF. The amount of human LF expressed by *S. cerevisiae* was estimated to be 3% to 5% of the total cellular protein. However, only cells carrying pLFISlO secreted significant amounts of human LF into the medium. The results revealed that approximately 5% to 7% of the total hLF was secreted, with the majority remaining intracellular or attached to membranes and the cell wall. Although the precursor hLF contains the invertase signal sequence of yeast, cells might not be able to secrete mammalian proteins as effectively as their own secretory proteins. Paramasivam et al. [66] successfully expressed intact equine LF in *P. pastoris* with a yield of 16.5 mg/l and confirmed that the product had a reasonable spatial conformation via circular dichroism spectroscopy. bLF was expressed intracellularly in *P. pastoris* KM71-H, with a final production of 3.5 g/l, and the expression product had a significant inhibitory effect on the bacteria [67]. This work highlighted the amount of LF produced, but it was difficult to industrialize due to the complexity of the purification process. KM71-H cells lyse the cell body and refold the inclusion body. Other reports have shown that goat LF, Tibetan sheep LF, and yak LF are expressed at levels of 2 mg/l, 60 mg/l, and 40 mg/l, respectively, in *P. pastoris* [68,69]. Importantly, the AOX1 promoter must be induced by methanol to ensure that the expression system of *P. pastoris* has safety risks in terms of production and application.

### Filamentous mold as a host to express LF

Filamentous molds have many advantages as cellular factories for protein production. Molds have strong protein expression abilities and secretion pathways and can perform posttranslational processing, including polypeptide chain shear, glycosylation, disulfide bond formation, and misfolded protein clearance [70,71]. In addition, mold also has strong survivability, which makes it superior to the heterologous “toxic protein” expression system represented by *E. coli* and yeast. Hence, mold is more suitable as a chassis cell to produce LF, a “toxic protein” with broad-spectrum antibacterial properties. However, the long growth cycle of mold limits its large-scale application in industry.

As the first mold used to express human LF, *Aspergillus nidulans* was produced in a yield of only 5 mg/l [70]. The Conneely group was the first to use *Aspergillus oryzae* as the host to express human LF at an expression level of 25 mg/l. The recombinant protein produced had the same molecular weight, immune reactivity, and iron ion binding ability as the naturally extracted LF [20]. Furthermore, the team fused human LF with glucose amylase and expressed it in *Aspergillus awamori*, with an expression yield exceeding 2 g/l in 1995, after which it was cleaved by the endogenous peptidase KEX-2 to obtain mature LF [71]. Because there was no difference in efficacy compared with the placebo group in the phase III clinical trial, the recombinant protein drug ultimately failed to enter the market successfully. However, this study still allows us to understand the powerful ability of mold expression systems to produce heterologously toxic proteins.

### Cell lines as hosts for the expression of LF

The process of synthesizing LF in mammalian cells has the potential to yield products that exhibit a high degree of consistency in structure and function with natural proteins. Kruzel et al. [72] induced the expression of LF in Chinese hamster ovary (CHO) cells, and the yield reached 200 mg/l. The glycosylation machinery of the CHO expression system bears a striking resemblance to that of the human body [73]. However, mammalian culture has relatively high technical and economic requirements and is susceptible to contamination or the ability to carry human pathogens. Furthermore, the use of mammalian cells expressing proteins in large-scale bioreactors is still limited. However, the Singapore biotechnology company TurtleTree announced that it has produced recombinant LF from breast cells and plans to launch a commercial product in the United States. This indicates that the problem of using mammalian cells as hosts for LF production can be overcome.

In addition to mammalian cell lines, transgenic animals are created by inserting a gene into the genome of an animal, thereby enabling the transfer of foreign genes to subsequent generations in a stable manner. The exocrine organ, the mammary gland, is generally the preferred site for the expression of heterologous genes. Milk does not enter the body’s circulation and affects the physiological metabolism of the host, and the recombinant protein has stable biological activity because it is fully processed and modified during secretion. Currently, human LF has been successfully expressed in transgenic mice, goats, pigs, and cattle [74]. However, the production of LF through microbial fermentation technology is the main focus of synthetic biological systems.

## Prospects

As a multifunctional protein, LF is involved in many important physiological processes. LF has broad-spectrum antibacterial, antiviral, and immunomodulatory effects and is used in the infant formula milk powder, nutritional supplement, and cosmetic industries. However, it has been difficult to obtain a large amount of biologically active LF. Therefore, a multidisciplinary strategy including molecular biology, protein engineering, fermentation optimization, downstream processing, and analytical characterization is needed to maximize LF production in synthetic biological systems. In addition, considering synthetic biological systems, yeast and mold are more competitive choices than are mammalian cells, which have high culture costs, and transgenic animals. Synthetic biological systems, such as *P. pastoris*, produce LF with appropriate antimicrobial activity in high yields. However, the toxicity of LF to the expression host is still an obstacle for its expression. We should consider reducing the toxicity of LF to the host during fermentation, which may greatly increase protein expression. We should consider rebuilding the LF transport system of the expression host to ensure that the synthesized LF is quickly secreted into the extracellular space. We should consider modifying chassis cells to tolerate higher concentrations of LF to obtain high-yielding strains of commercial value. In addition, we should also consider knocking out key proteases that can degrade LF in the host. Overall, it will be important to explore efficient, safe, and mass-produced synthetic systems of LF in the future.
